# Intensifying Electron Utilization by Surface-Anchored Rh Complex for Enhanced Nicotinamide Cofactor Regeneration and Photoenzymatic CO_2_ Reduction

**DOI:** 10.34133/2021/8175709

**Published:** 2021-02-18

**Authors:** Yuqing Cheng, Jiafu Shi, Yizhou Wu, Xueying Wang, Yiying Sun, Ziyi Cai, Yu Chen, Zhongyi Jiang

**Affiliations:** ^1^Key Laboratory for Green Chemical Technology of Ministry of Education, School of Chemical Engineering and Technology, Tianjin University, 92 Weijin Road, Nankai District, Tianjin 300072, China; ^2^Collaborative Innovation Center of Chemical Science and Engineering (Tianjin), 92 Weijin Road, Nankai District, Tianjin 300072, China; ^3^School of Environmental Science & Engineering, Tianjin University, 92 Weijin Road, Nankai District, Tianjin 300072, China; ^4^State Key Laboratory of Biochemical Engineering, Institute of Process Engineering, Chinese Academy of Sciences, Beijing 10090, China

## Abstract

Solar-driven photocatalytic regeneration of cofactors, including reduced nicotinamide adenine dinucleotide (NADH), reduced nicotinamide adenine dinucleotide phosphate (NADPH), and reduced flavin adenine dinucleotide (FADH_2_), could ensure the sustainable energy supply of enzymatic reactions catalyzed by oxidoreductases for the efficient synthesis of chemicals. However, the elevation of cofactor regeneration efficiency is severely hindered by the inefficient utilization of electrons transferred on the surface of photocatalysts. Inspired by the phenomenon of ferredoxin-NADP^+^ reductase (FNR) anchoring on thylakoid membrane, herein, a homogeneous catalyst of rhodium (Rh) complex, [Cp∗Rh(bpy)H_2_O]^2+^, was anchored on polymeric carbon nitride (PCN) mediated by a tannic acid/polyethyleneimine (TA/PEI) adhesive layer, acquiring PCN@TA/PEI-Rh core@shell photocatalyst. Illuminated by visible light, electrons were excited from the PCN core, then transferred through the TA/PEI shell, and finally captured by the surface-anchored Rh for instant utilization during the regeneration of NADH. The TA/PEI-Rh shell could facilitate the electron transfer from the PCN core and, more importantly, achieved ~1.3-fold elevation of electron utilization efficiency compared with PCN. Accordingly, the PCN@TA/PEI-Rh afforded the NADH regeneration efficiency of 37.8% after 20 min reaction under LED light (405 nm) illumination, over 1.5 times higher than PCN with free Rh. Coupling of the NADH regeneration system with formate dehydrogenase achieved continuous production of formate from carbon dioxide (CO_2_). Our study may provide a generic and effective strategy to elevate the catalytic efficiency of a photocatalyst through intensifying the electron utilization.

## 1. Introduction

In living organisms, enzymes are the “catalytic machine” to trigger biological reactions for ensuring the steady implementation of metabolic processes. The features of such “catalytic machine” combined with the reactivity of synthetic chemical catalyst have spawned the field of chemoenzymatic catalysis, which can be categorized into three reaction types, i.e., sequential reactions, concurrent reactions, and cooperative reactions [1].

As a typical cooperative chemoenzymatic catalysis, photoenzymatic catalysis inherits the light harvesting capability of semiconductor photocatalyst and high activity/selectivity of enzyme, which can convert ubiquitous and clean solar energy into chemical energy. Till now, the photoenzymatic catalysis has been applied for carbon dioxide (CO_2_) reduction [2–4], hydrogen/oxygen (H_2_/O_2_) evolution [5–7], and biomass conversion [8, 9]. During the photoenzymatic process, the nicotinamide cofactor is predominantly used as “energy currency” to coordinate photocatalysis and enzyme catalysis. Meanwhile, the photocatalytic regeneration of nicotinamide cofactor usually exhibits much lower rate (10^−3^-10^−2^ s^−1^) compared with the enzymatic reaction (10^2^-10^3^ s^−1^) [10]. Design and preparation of high-performance photocatalyst materials is thus urgently required for acquiring an improved nicotinamide cofactor regeneration efficiency.

In general, the photocatalytic process includes three main steps: charge generation, charge transfer, and charge utilization [11]. The excitation of photocatalyst by light generates holes and electrons. Usually, only part excited electrons are transferred to surface of photocatalyst and then utilized by the catalytic center to trigger the reduction reaction. To elevate the photocatalytic efficiency, the intensification of electron generation and/or electron transfer has been actively explored, which could be realized by engineering the regular structure [12], increasing the specific surface area [13, 14], optimizing the conduction interface [15], designing heterojunctions [16–18], and so on. Albeit grand achievements in the elevation of either electron generation or electron transfer, the intensification of electron utilization is rarely reported [19]. For a photocatalytic reaction, particularly photocatalytic nicotinamide cofactor regeneration, the electron utilization is severely hindered by the longer electron transfer and lower contact probability between the photocatalyst surface and the free catalytic center ([Cp∗Rh(bpy)H_2_O]^2+^, denoted as Rh). Meanwhile, the presence of free catalytic center further increases subsequent difficulty and cost for target product recovery. Therefore, it is imperative to engineer the photocatalyst with enhanced electron utilization efficiency for nicotinamide cofactor regeneration.

**Figure 1:**
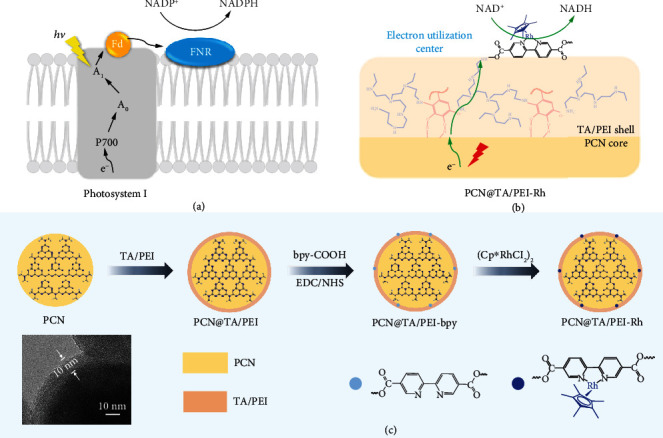
(a) Schematic diagram of NADPH regeneration in natural photosynthesis. (b) Schematic diagram of photocatalytic NADH regeneration with PCN@TA/PEI-Rh. (c) Schematic preparation of PCN@TA/PEI-Rh core@shell photocatalyst (the bottom left part was the TEM image of SiO_2_@TA/PEI).

In nature photosynthesis, ferredoxin-NADP^+^ reductase (FNR), as part of thylakoid membrane anchoring on the surface (Figure 1(a)), is the electron utilization center during the regeneration of reduced nicotinamide adenine dinucleotide phosphate (NADPH) [20]. Under visible light irradiation, electrons are generated and transferred to protein complex photosystem I (PS I) through an electron transfer chain [11] and stored in ferredoxin (Fd), which is directly connected with three subunits of PS I [21]. Then, the electrons are transferred from a 2Fe-2S cluster of Fd to flavin adenine dinucleotide (FAD) prosthetic group of FNR, the ultimate destination in electron transfer chain, for utilization in the regeneration of NADPH. In particular, the distance between 2Fe-2S cluster and FAD is extremely short, which relies on the electrostatic interactions formed through salt bridges. The electron transfer distance from photosensitizer to the electron utilization center is shortened for nanoconfinement between Fd and FNR, which elevates the electron utilization. The unique mode of anchored FNR guarantees about 100% of the inherent quantum efficiency for natural photosynthesis [22].

Inspired by the structure and function of FNR anchored on thylakoid membrane, a tannic acid/polyethyleneimine (TA/PEI) nanoshell anchored with [Cp∗Rh(bpy)H_2_O]^2+^ (Rh) is formed on the surface of polymeric carbon nitride (PCN) through polyphenol-induced surface adhesion process [23–25]. The acquired core@shell photocatalyst was denoted as PCN@TA/PEI-Rh (Figure 1(b)). The PCN, a typical metal-free semiconductor material [26], is chosen as photoresponsive core to generate electrons under visible light illumination, similar to PS I on thylakoid membrane. Then, the photoexcited electrons are transferred and utilized by the Rh immobilized on TA/PEI nanoshell for nicotinamide cofactor regeneration, similar to FNR anchored on thylakoid membrane. The surface-anchored Rh intensifies utilization of photoexcited electrons arisen from the shortened electron transfer distance between Rh and PCN, which can also simplify nicotinamide cofactor regeneration system. The content of immobilized Rh can be facilely controlled by altering the chemical compositions of TA/PEI nanoshell. The photoenzymatic conversion of carbon dioxide (CO_2_) could be realized through coupling PCN@TA/PEI-Rh with formate dehydrogenase (FDH). Combined with the controllability and mild condition of polyphenol-induced adhesion method, our study may offer a generic strategy to improve the photocatalytic efficiency via intensifying the electron utilization.

## 2. Results

### 2.1. Preparation and Characterizations of PCN@TA/PEI-Rh Core@Shell Photocatalyst

In our study, the PCN@TA/PEI-Rh core@shell photocatalyst was prepared through anchoring an Rh complex ([Cp∗Rh(bpy)H_2_O]^2+^, a molecular catalyst for selective hydrogenation of pyridine ring) on polymeric carbon nitride (PCN) mediated by a polyphenol-inspired adhesive layer [24]. The general preparation process is demonstrated in Figure 1(c). Briefly, TA, a typical polyphenol, was adhered on the PCN surface and further crosslinked with PEI triggered by a pH change, forming the PCN@TA/PEI particle. Then, the coupling reaction between PEI moiety in the TA/PEI nanoshell and bipyridine carboxylate (bpy-COOH) occurred with the addition of N-hydroxysuccinimide (NHS) and 1-(3-dimethylaminopropyl)-3-ethylcarbodiimide hydrochloride (EDC), where PCN@TA/PEI-bpy particle was obtained. Finally, PCN@TA/PEI-Rh core@shell photocatalyst was obtained *via* the coordination of bipyridine and (Cp∗RhCl_2_)_2_ (Figure S1). As shown in Figure S2a, PCN@TA/PEI-Rh exhibited a typical bulky structure, where C, N, O, and Rh elements were uniformly distributed (Figure S2b–e). Particularly, the presence of O and Rh element preliminary validated the existence of TA molecules and Rh complex on the surface of PCN core. In order to visually observe the thickness of TA/PEI nanoshell, SiO_2_ core with a regular sphere structure was used instead of PCN to prepare SiO_2_@TA/PEI. Clearly, the TA/PEI nanoshell with a thickness of ~10 nm was observed in the TEM image (Figure 1(c)).

**Figure 2:**
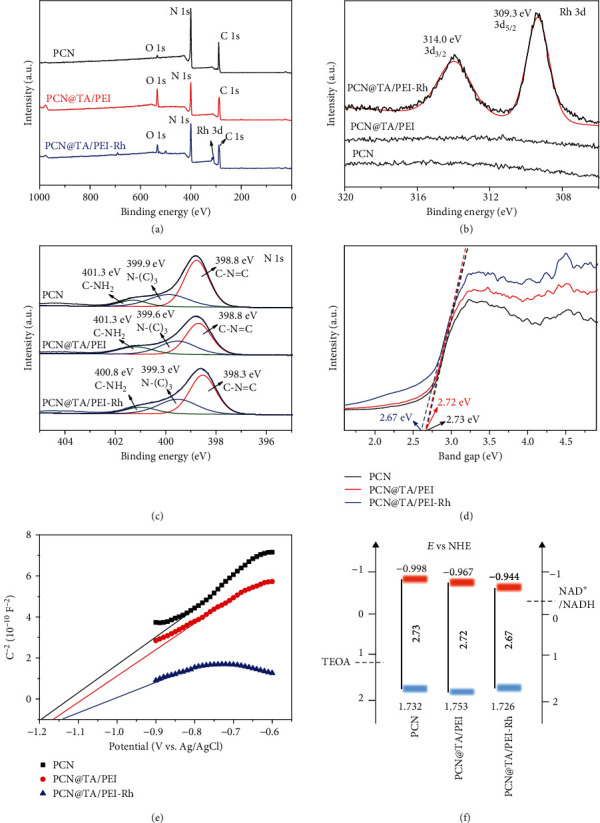
(a) XPS survey spectra of PCN, PCN@TA/PEI, and PCN@TA/PEI-Rh. High-resolution XPS (b) Rh 3d and (c) N 1s spectra of PCN, PCN@TA/PEI, and PCN@TA/PEI-Rh. (d) Calculated band gap patterns based on UV-vis diffuse reflectance spectra. (e) Mott-Schottky plots of PCN, PCN@TA/PEI, and PCN@TA/PEI-Rh. (f) Conduction (orange) and valence (blue) band-edge positions of PCN, PCN@TA/PEI, and PCN@TA/PEI-Rh.

Subsequently, the chemical and physical compositions of PCN@TA/PEI-Rh were examined by Fourier transform infrared spectroscopy (FTIR), X-ray photoelectron spectroscopy (XPS), and X-ray powder diffraction (XRD). Commonly, PCN prepared by thermal polycondensation of melamine showed a graphite-like lamellar structure, of which the PCN layers had a triazine ring structure and co-ssembled *via* hydrogen bonds and *π* − *π* interaction. As shown in Figure S3, PCN exhibited two strong peaks at 12.9° and 27.4° in the XRD pattern, which were assigned to the *π* − *π* conjugated structures and tri-*s*-triazine structures of PCN, respectively. The existence of triazine ring in PCN was also validated by its absorption band at 806 cm^−1^ in the FTIR spectrum (Figure S4). Moreover, the absorption bands at 3000-3500 cm^−1^ and 1234-1635 cm^−1^ were, respectively, corresponded to N-H stretching and C-N/C=N stretching, further indicating the successful preparation of PCN. As for PCN@TA/PEI and PCN@TA/PEI-Rh, the absorption bands that should be assigned to Ar-OH (1200 cm^−1^) and C=O (1717 cm^−1^) in the TA molecules were not observed due to the interference of the absorption bands of PCN. Fortunately, the presence of O 1s peak in the XPS survey spectra of PCN@TA/PEI and PCN@TA/PEI-Rh (Figure 2(a)) verified the existence of TA molecules on PCN. The anchoring of Rh on PCN was also validated by the presence of Rh 3d peak in the XPS survey spectra of PCN@TA/PEI-Rh (Figure 2(b)). Specifically, the peaks appeared at 314.0 eV and 309.3 eV were assigned to the binding energies of Rh 3d_2/3_ and Rh 3d_5/2_, respectively (Figure 2(b)). High-resolution C 1s XPS spectra of PCN@TA/PEI-Rh (Figure S5) could be deconvoluted to four peaks at 287.9 eV, 286.2 eV, 285.3 eV, and 284.5 eV. The main peaks at 287.9 eV and 284.5 eV were assigned to the N-C=N bond and C-C bond of PCN, respectively. Two new peaks at 286.2 and 285.3 eV were attributed to the C atom of C-O bond and C-N bond of TA and PEI, respectively. For O 1s spectra (Figure S6), two new peaks of PCN@TA/PEI-Rh at 532.2 eV and 530.8 eV were clearly observed, corresponding to O of C=O bond and C-O bond in TA, respectively. The N 1s spectra of PCN, PCN@TA/PEI, and PCN@TA/PEI-Rh (Figure 2(c)) all showed the characteristic peaks of C-NH_2_, N-(C)_3_, and C-N=C. In particular, the characteristic peaks at 400.8 eV, 399.3 eV, and 398.3 eV of PCN@TA/PEI-Rh slightly shifted compared to the characteristic peaks at 401.3 eV, 399.6 eV, and 398.8 eV of PCN@TA/PEI, which revealed the existence of chemical interaction between Rh and TA/PEI nanoshell.

The band structures of PCN, PCN@TA/PEI, and PCN@TA/PEI-Rh were then characterized by XPS valence band (VB) spectra, the Mott-Schottky plot, and UV-vis diffuse reflection spectroscopy (DRS). As shown in Figure S7, the absorption edge of PCN@TA/PEI-Rh (482 nm) was wider than that of PCN (456 nm) and PCN@TA/PEI (464 nm). The PCN@TA/PEI-Rh exerted narrower band gap of 2.67 eV in the UV-vis DRS spectra (Figure 2(d)), indicating that the TA/PEI nanoshell grafted with Rh widened the visible light absorption range of PCN. The conduction band (CB) values of PCN, PCN@TA/PEI, and PCN@TA/PEI-Rh detected by Mott-Schottky plots (Figure 2(e)) were -1.195 V, -1.164 V, and -1.141 V vs. Ag/AgCl, pH = 7.0, respectively (equal to -1.195 V, -1.164 V, and-1.141 V vs. NHE, pH = 7.0). According to band gap and CB analysis, the VB potentials of PCN, PCN@TA/PEI, and PCN@TA/PEI-Rh were calculated to 1.732 V, 1.753 V, and 1.726 V (vs. NHE, pH = 7.0), respectively. It should be noted that the redox potential of NAD^+^/NADH was -0.32 V (vs. NHE, pH = 7.0) [11], which was lower than the CB of PCN, PCN@TA/PEI, and PCN@TA/PEI-Rh (Figure 2(f)). This indicated that PCN, PCN@TA/PEI, and PCN@TA/PEI-Rh could be able to regenerate NADH.

The transfer and utilization processes of photoexcited electrons of PCN and PCN@TA/PEI-Rh were elucidated by photoelectrochemical analysis. As shown in Figure S8, PCN@TA/PEI-Rh excited by light showed ~150% elevation of the carrier density by contrast with PCN. This suggested the transfer of more photoexcited electrons from bulk to surface. More electrons could then be readily used. As for the PL spectra, the PCN@TA/PEI-Rh displayed a much lower absorption peak at 470 nm compared with PCN (Figure S9), indicating a lower electron-hole recombination. We could then conjecture that more photoexcited electrons were captured by the anchored Rh, which was beneficial for improving the utilization efficiency of transferred electrons. Hence, we further calculated the Hirshfeld charge of PCN@TA/PEI and PCN@TA/PEI-Rh through DFT. As shown in Figure S10, to simplify the model, bpy-H and bpy-Rh-H were used to represent PCN@TA/PEI and PCN@TA/PEI-Rh, respectively, for examining the role of Rh. Accordingly, two models of bpy-H and bpy-Rh-H were optimized by Gaussian 03 program. Calculated with Multiwfn, the Hirshfeld charge of the additional H atom in bpy-Rh-H was -0.06 while that in bpy-H is more positive (0.15). The calculation indicated that more electrons were enriched on the surface-anchored Rh, so that more electrons could be utilized for NADH regeneration.

**Figure 3:**
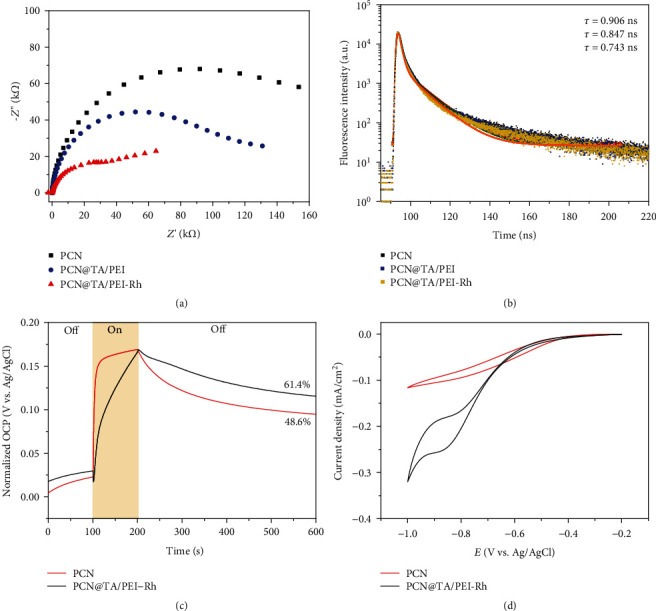
(a) EIS Nyquist plots of PCN, PCN@TA/PEI, and PCN@TA/PEI-Rh in 0.1 M Na_2_SO_4_ without illumination. (b) Time-resolved transient PL spectra of PCN, PCN@TA/PEI, and PCN@TA/PEI-Rh. (c) Open-circuit potential of PCN and PCN@TA/PEI-Rh as a function of time. (d) Cyclic voltammetry curve of PCN and PCN@TA/PEI-Rh at a scan rate of 5 mV s^−1^.

To figure out whether the TA/PEI moiety affects the electron transfer or utilization behavior, the electron transfer behaviors of PCN, PCN@TA/PEI, and PCN@TA/PEI-Rh were monitored by EIS Nyquist plots and time-resolved transient PL spectra. As shown in Figure 3(a), the curve radius of PCN@TA/PEI-Rh was less than PCN and PCN@TA/PEI. That illustrated the lower interfacial electron transfer resistance of PCN@TA/PEI-Rh, which could promote electron transfer rate for PCN@TA/PEI-Rh. In the time-resolved transient PL spectra (Figure 3(b)), the decay curves of PCN, PCN@TA/PEI, and PCN@TA/PEI-Rh were fitted by the following biexponential equation:

(1) $$\begin{array}{c} \text{I}\left(t\right)=A+B_{1}\exp\left(-\frac{\text{t}}{\tau_{1}}\right)+B_{2}\exp\left(-\frac{t}{\tau_{2}}\right), \end{array}$$

where *B*_1_ and *B*_2_ were the corresponding amplitudes and *τ*_1_ and *τ*_2_ were the fluorescent lifetimes. The average fluorescent lifetime was described in the following equation:

(2) $$\begin{array}{c} \tau =\frac{{\text{B}}_{1}\tau_{1}^{2}+B_{2}\tau_{2}^{2}}{B_{1}\tau_{1}+B_{1}\tau_{2}}. \end{array}$$

With parameters in Table S1, the fluorescence lifetime of PCN, PCN@TA/PEI, and PCN@TA/PEI-Rh could be calculated to 0.906 ns, 0.847 ns, and 0.743 ns, respectively, further evidencing the electron transfer from PCN to Rh through TA/PEI nanoshell. All above results evidenced that the TA/PEI moiety did not alter the electron transfer or utilization process, whereas the anchoring of Rh captured more electrons for further utilization. We then quantitatively analyzed the electron utilization efficiency (EUE) by the methods as reported in our previous work [15]. As shown in Figure 3(c), the open-circuit potential (OCP) of PCN and PCN@TA/PEI-Rh was measured. During the illumination time of 100 s, the voltage increased with the accumulation of holes. After irradiation, the reduced voltage indicated the recombination of electrons and holes. The EUE of PCN@TA/PEI-Rh was calculated to 61.4% with the following equation3, achieved ~1.3-fold elevation compared with that of PCN (48.6%).

(3) $$\begin{array}{c} \text{EUE}=\left(\frac{V_{2}-V_{0}}{V_{1}-V_{0}}\right)\times 100\%, \end{array}$$

where *V*_0_, *V*_1_, and *V*_2_ are the OCPs at 100, 200, and 600 s, respectively. In the cyclic voltammetry curve of PCN and PCN@TA/PEI-Rh (Figure 3(d)), a wider closed curve of PCN@TA/PEI-Rh was detected and a redox peak at -0.849 V was observed (vs. Ag/AgCl), indicating that the redox reaction occurred when electrons were transferred from PCN to Rh.

### 2.2. Nicotinamide Cofactor Regeneration and Photoenzymatic CO_2_ Conversion

**Figure 4:**
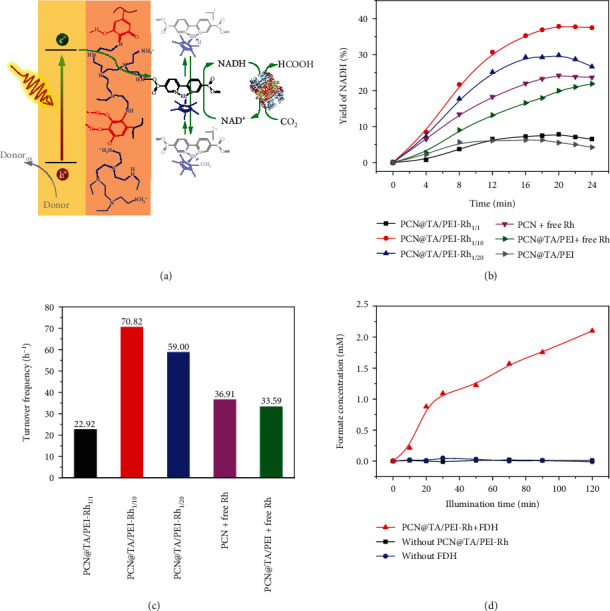
(a) Scheme of nicotinamide cofactor regeneration system. (b) The efficiency of NADH catalyzed by PCN@TA/PEI-Rh_a/b_ with different [TA]/[PEI], PCN, and PCN@TA/PEI with free Rh system. (c) The turnover frequency (TOF) of PCN@TA/PEI-Rh_1/1_, PCN@TA/PEI-Rh_1/10_, PCN@TA/PEI-Rh_1/20_, PCN, and PCN@TA/PEI with free Rh system. (d) Photoenzymatic conversion of CO_2_ enabled by PCN@TA/PEI-Rh. Reaction conditions: (b) [photocatalyst] = 0.5 mg mL^−1^, PBS buffer (pH 7.0, 100 mM), [NAD^+^] = 1 mM, [free Rh] = 0.028 mM, [TEOA] = 400 mM, visible light illumination (*λ* = 405 *nm*), volume = 2 mL, and room temperature; (d) [PCN@TA/PEI − Rh] = 0.5 mg mL^−1^, [FDH] = 2 mg mL^−1^, [NAD^+^] = 5 mM, and [CO_2_] = 0.3 MPa.

During the photoenzymatic process, NADH (reduced nicotinamide adenine dinucleotide, a typical nicotinamide cofactor) is frequently used as the “energy currency” to bridge photocatalysis and enzyme catalysis [27]. The efficient regeneration of NADH by photocatalyst is thus urgently required. As shown in Figure 4(a), the [Cp∗Rh(bpy)H_2_O]^2+^ (Rh) was used as electron mediator and triethanolamine (TEOA) was used as electron donor to perform photocatalytic NADH regeneration. Under visible light illumination, the photoexcited electrons on PCN were transferred to Rh on the TA/PEI nanoshell and then triggered the regeneration of NADH, whereas the holes on PCN were quenched by TEOA to suppress the electron-hole recombination. The efficiency of regenerated NADH was calculated by detecting the absorbance of NADH at 340 nm with UV-visible spectroscopy and verified by enzyme assay.

In our study, the chemical structures of nanoshell were firstly optimized through changing the ratios of TA concentration to PEI concentration ([TA]/[PEI]) and coupling reaction time (*X*) of PEI and bpy-COOH to investigate their effect on NADH regeneration efficiency. Specifically, three samples of PCN@TA/PEI-Rh with different [TA]/[PEI] were prepared. As shown in Figure 4(b), the optimized NADH regeneration efficiencies of three PCN@TA/PEI-Rh were all presented at 20 min. With the increase of [TA]/[PEI] from 1/1 to 1/10, the optimized NADH regeneration efficiency increased from 7.8% to 37.8%. This was probably due to the increase of Rh content from 0.020 mM to 0.028 mM as detected by inductively coupled plasma spectrometer (ICP-OES, Figure S11). Subsequently, the NADH regeneration efficiency further slightly decreased from 37.8% to 29.8% with the increase of [TA]/[PEI] from 1/10 to 1/20 as evidenced by the slight decrease of Rh content from 0.028 mM to 0.027 mM (Figure S11). Then, the photocatalytic performance of PCN@TA/PEI-Rh was optimized by altering the *X* of PEI and bpy-COOH. The extension of *X* was beneficial to the efficiency of photocatalytic NADH regeneration (Figure S12). Specially, the NADH regeneration efficiency unaltered when *X* was over 48 h, indicating that the coupling reaction between amino and carboxyl groups was completed at 48 h.

Based on above analysis, the NADH regeneration efficiencies of different photocatalysts with free Rh (the content of free Rh was the same as that of PCN@TA/PEI-Rh_1/10_) and surface-anchored Rh were measured. The reaction setup is shown in Figure S13. As shown in Figure 4(b), the optimized NADH regeneration efficiency of PCN@TA/PEI-Rh (37.8%) was 156% higher than that of PCN with free Rh (24.2%), which should be arisen by the higher EUE. Meanwhile, the turnover frequency (TOF) of PCN@TA/PEI-Rh_1/1_, PCN@TA/PEI-Rh_1/10_, PCN@TA/PEI-Rh_1/20_, PCN, and PCN@TA/PEI with free Rh system was calculated as shown in Figure 4(c). By contrast, the PCN@TA/PEI-Rh_1/10_ showed TOF of 70.82 h^−1^ based on Rh, which is, to the best of our knowledge, the highest rate among the reported photocatalytic NADH regeneration (Table S2).

**Figure 5:**
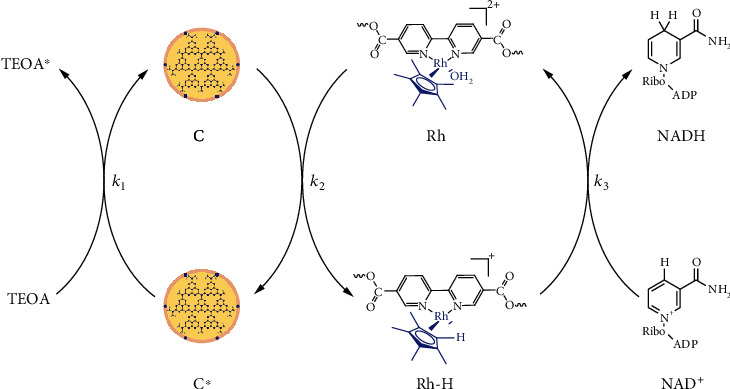
Reaction mechanism of photocatalytic NADH regeneration with PCN@TA/PEI-Rh.

To further in-depth understand the NADH regeneration process, the reaction kinetics were analyzed based on the experiment measured at different NAD^+^ concentrations (0.1 mM-0.5 mM) (Table S3) [28]. In general, the photocatalytic NADH regeneration process with PCN@TA/PEI-Rh could be categorized into three primary steps (Figure 5): (1) redox reaction between TEOA and catalyst (donated as C∗, the catalyst in its oxidized form); (2) redox reaction between catalyst and Rh; and (3) redox reaction between Rh-H (the Rh in its hydride form) and NAD^+^. Given the above conditions, the mathematical equations relating *r*_NADH_(*d*[NADH]/*dt*) to [NAD^+^] are shown in the following equations, imposing the steady-state concentration to C and Rh (as detailed in Supplementary Materials).

(4) $$\begin{array}{c} r_{\text{NADH}}=\frac{d\left[\text{NADH}\right]}{dt}=k_{3}\left[\text{Rh}-\text{H}\right]\left[{\text{NAD}}^{+}\right], \end{array}$$

(5) $$\begin{array}{c} k_{1}k_{3}\left[\text{TEOA}\right]\left[{\text{NAD}}^{+}\right]\left[\text{Rh}-\text{H}\right]=\left({\text{C}}_{\text{Rh}}-\left[\text{Rh}-\text{H}\right]\right)\left(k_{1}k_{2}{\text{C}}_{\text{C}}\left[\text{TEOA}\right]-k_{2}k_{3}\left[{\text{NAD}}^{+}\right]\left[\text{Rh}-\text{H}\right]\right). \end{array}$$

Substituting the experimental data *r*_NADH_ versus [NAD^+^] into Equations (4) and (5), *k*_1_, *k*_2_, and *k*_3_ were obtained (Table 1). The result confirmed the superiority of PCN@TA/PEI-Rh as evidenced by the higher capability of surface-anchored Rh to utilize electrons (*k*_2_ of PCN@TA/PEI-Rh was 20 times larger than that for PCN) and lower density of holes to inhibit recombination (*k*_1_ of PCN@TA/PEI-Rh was 10 times larger than that of PCN). As for the reaction between Rh-H and NAD^+^ (*k*_3_ in Table 1), PCN@TA/PEI-Rh was inactive than PCN arising from the transition from liquid-liquid homogeneous phase to solid-liquid heterogeneous phase between Rh and NAD^+^, but the difference is less critical (*k*_3_ was 3.5 times smaller than for PCN).

Finally, the recycling stability of PCN@TA/PEI-Rh was investigated. After 5 cycles, ~52% activity of NADH regeneration efficiency was retained (Figure S14). To assess the enzymatic activity of regenerated NADH, formate dehydrogenase (FDH) was coupled with PCN@TA/PEI-Rh for photoenzymatic conversion of CO_2_ into formate [29]. As shown in Figure 4(d), the PCN@TA/PEI-Rh and FDH-coupled system produced 2.1 mM formate in 120 min, and each NADH was used 2.32 times for formate production (calculated with Equation (8)). By contrast, in the absence of PCN@TA/PEI-Rh or FDH, no accumulation of formate was detected.

## 3. Discussion

In summary, the PCN@TA/PEI-Rh core@shell photocatalyst was prepared by anchoring Rh complex on the PCN core through polyphenol-induced adhesion method for light-driven NADH regeneration and photoenzymatic CO_2_ conversion. Under visible light illumination, the PCN core was excited to generate electrons, whereas the surface-anchored Rh on TA/PEI nanoshell acted as the electron utilization center toward NADH regeneration. The electron utilization was remarkably fortified by the surface-anchored Rh as validated by open-circuit potential, which could be regulated by changing the [TA]/[PEI] ratio and coupling reaction time of PEI and bpy-COOH. Under optimal condition, the PCN@TA/PEI-Rh exerted a ~1.3-fold elevation in EUE compared with the system where Rh was in free form, leading to a photocatalytic NADH regeneration efficiency of ~37.8%. Photoenzymatic conversion of CO_2_ could then be realized by coupling PCN@TA/PEI-Rh with formate dehydrogenase (FDH). Hopefully, our study may provide a generic strategy to intensify electron utilization for enhanced photocatalytic performance.

## 4. Materials and Methods

### 4.1. Materials

Pentamethylcyclopentadienyl rhodium (III) chloride dimer ((Cp∗RhCl_2_)_2_) and triethanolamine (TEOA) were purchased from Aladdin Industrial Corporation (Shanghai, China). Tannic acid (TA), N-hydroxysuccinimide (NHS), and 2,2′-bipyridine-5,5′-dicarboxylic acid were purchased from J&K Chemical (Shanghai, China). *β*-Nicotinamide adenine dinucleotide phosphate sodium salt hydrate (NAD^+^), formate dehydrogenase (FDH) from *Candida boidinii*, melamine, polyethyleneimine (PEI; average Mw ∼2000 Da), tris(hydroxymethyl)aminomethane (Tris), and silica (SiO_2_ powder, 0.2-0.3 *μ*m) were purchased from Sigma-Aldrich (St. Louis, USA). 1-(3-Dimethylaminopropyl)-3-ethylcarbodiimide hydrochloride (EDC) was purchased from TCI Chemical (Shanghai, China). All other materials were used without further purification.

### 4.2. Preparation of PCN

Polymeric carbon nitride (denoted as PCN) was synthesized by the condensation reaction of melamine with a one-step heated treatment. Firstly, melamine was weighed in a small crucible, which was sealed and wrapped with three layers of tin foil. Then, the crucible was stayed in a muffle furnace at 550°C for 4 h, of which the temperature was increased to 550°C at a rate of 5°C min^−1^. Finally, the yellow PCN was obtained after the temperature dropped to room temperature.

### 4.3. Preparation of PCN@TA/PEI

Tannic acid/polyethyleneimine (TA/PEI) nanoshell was prepared according to our previous study [25]. Firstly, the mixed buffer solution (pH 6.0) containing 50 mM Tris-HCl and 0.5 mg mL^−1^ TA was prepared. The PCN (80 mg) as synthesized above was added to it with vigorous stirring for 1 min. Then, different concentrations of PEI (0.5 mg mL^−1^, 5.0 mg mL^−1^, and 10.0 mg mL^−1^) were mixed with above solution. The pH was further adjusted to 8.0, and the mixed solution was stirred for 1 h. Finally, the PCN@TA/PEI was obtained after centrifugation and washing for three times with water.

### 4.4. Preparation of PCN@TA/PEI-Rh

Firstly, the mixed buffer solution (pH 7.0) containing 50 mM phosphate-buffered saline (PBS) and 10 mM 2,2′-bipyridine-5,5′-dicarboxylic acid was prepared. The PCN@TA/PEI as prepared above was added to it with stirring. Next, EDC and NHS were added to the above solution with both final concentrations of 10 mM. After the pH was adjusted to 7.0, the mixed solution was stirred for 48 h. The precipitate was obtained, centrifuged at 6000 rpm, and washed for three times with water. Then, the previously collected precipitate was added to mixed solution (pH 7.0) with shaking for 24 h, which contained 50 mM PBS buffer and 1 mM (Cp∗RhCl_2_)_2_. Finally, the PCN@TA/PEI-Rh was obtained, centrifuged, and washed three times with water.

### 4.5. Preparation of SiO_2_@TA/PEI

Firstly, the mixed buffer solution (pH 6.0) containing 50 mM Tris-HCl and 0.5 mg mL^−1^ TA was prepared. The SiO_2_ (80 mg) was added to it with vigorous stirring for 1 min. Then, 5.0 mg mL^−1^ PEI was mixed with above solution. The pH was further adjusted to 8.0, and the mixed solution was stirred for 1 h. Finally, the SiO_2_@TA/PEI was obtained after centrifugation and washing for three times with water.

### 4.6. Synthesis of [Cp∗Rh(bpy)H_2_O]^2+^

Pentamethylcyclopentadienyl rhodium bipyridine ([Cp∗Rh(bpy)(H_2_O)]^2+^) was prepared according to previous literature [30]. Firstly, 61.8 mg of (Cp∗RhCl_2_)_2_ was added into methanol (4 mL) to obtain a red suspension. Then, 31.2 mg of 2,2-bipyridine was mixed with the suspension, acquiring a transparent orange solution. At room temperature, after concentrating the solution to 1 mL, anhydrous ether (8 mL) was added so that [Cp∗Rh(bpy)Cl]Cl gradually precipitated out. The [Cp∗Rh(bpy)Cl]Cl was obtained followed by centrifugation and washing for three times with water. After drying in oven, [Cp∗Rh(bpy)Cl]Cl was dissolved in H_2_O to obtain [Cp∗Rh(bpy)H_2_O]^2+^ (denoted as Rh), which was stored at 4°C in darkness and utilized to catalyze reaction of NADH regeneration.

### 4.7. Photoelectrochemical Measurement

All photoelectrochemical measurements were performed by an electrochemical workstation (CHI660) with a three-electrode system. Typically, the reference electrode was Ag/AgCl, the counter electrode was platinum foil (0.5 × 0.5 cm^2^), and the prepared samples were used as the working electrodes. The working electrodes were prepared by dispersing samples (5 mg) into 2% Nafion solution (0.5 mL) and dropping it onto FTO glass electrode (2.0 × 1.5 cm^2^). After drying overnight in 40°C oven, the films on FTO glass electrode were divided into 1.0 × 1.5 cm^2^ for subsequent measurement. The 0.1 M Na_2_SO_4_ was used as electrolyte, and the light source was a 300 W xenon lamp. The initial voltage of the transient photocurrent and electrochemical impedance spectroscopy (EIS) was kept at -0.1 V.

### 4.8. Photocatalytic Regeneration of NADH

The photocatalytic NADH regeneration was proceeded in a quartz reactor (1.25 × 1.25 × 4.5 cm^3^) with reaction mixture (2 mL), which contained 0.5 mg mL^−1^ photocatalyst, 1 mM NAD^+^, 400 mM TEOA, and 100 mM PBS (pH = 8.0). Firstly, the mixture was hatched for 10 min without illumination, and then a 100 W LED lamp (405 nm) was used to activate photocatalyst for 24 min. The sample was taken every 4 min, of which the absorbance at 340 nm was detected by a UV-vis spectrophotometer (U-3010, Hitachi). On the basis of the detected absorbance with the following equation, we could calculate the concentration of regenerated NADH.

(6) $$\begin{array}{c} c=\frac{A}{Kb}, \end{array}$$

where *c* was the concentration of regenerated NADH, *A* was the detected absorbance at 340 nm of sample, *K* was an extinction coefficient of 6220 M^−1^ cm^−1^ [26], and *b* was the optical path of 1 cm.

The turnover frequency (TOF) of NADH based on Rh was calculated with the following equation:

(7) $$\begin{array}{c} \text{TOF}=\frac{c_{\text{NADH}}}{c_{\text{Rh}}t}, \end{array}$$

where *c*_NADH_ and *c*_Rh_ were the concentrations of regenerated NADH and [Cp∗Rh(bpy)H_2_O]^2+^ (Rh), respectively and *t* was 4 min (based on the 4-8 min of reaction).

### 4.9. Photoenzymatic Production of Formate from CO_2_

Photoenzymatic conversion of CO_2_ (0.3 MPa) was performed in a visible photocatalytic autoclave with 4 mL mixed suspension (37°C), which contained 0.5 mg mL^−1^ photocatalyst, 100 mM PBS (pH = 8.0), 5 mM NAD^+^, 400 mM TEOA, and 2 mg mL^−1^ formate dehydrogenase (FDH). A 100 W LED lamp (405 nm) was used as optical source for 120 min. After every 10 min illumination, the sample was taken and centrifuged. The concentration of formic acid in the supernatant was detected by previously reported chromogenic method [31].

According to the enzymatic reaction ${\text{CO}}_{2}+\text{NADH}\overset{\text{FDH}}{\to }{\text{HCOO}}^{-}+{\text{NAD}}^{+}$, NADH was consumed following the stoichiometric ratio during the reaction of formate production. The mole of regenerated NADH was the same as that of formate produced during the reaction. The times of individual NADH utilized in formate production were calculated with the following equation:

(8) $$\begin{array}{c} N_{\text{NADH}}=\frac{c_{\text{Formate}}}{c_{\text{NADH}}}, \end{array}$$

where *N*_NADH_ was the times of individual NADH used for formate production, *c*_Formate_ was the concentration of formate, and *c*_NADH_ was the concentration of NADH.

### 4.10. Characterizations

Fourier transform infrared (FTIR) spectra were conducted by a Nicolet-6700 spectrometer. X-ray powder diffraction (XRD) was collected on a Rigaku D/max 2500V/PC instrument, and the data was obtained in the range of 10-60° (2*θ*) at a rate of 5° min^−1^. Transmission electron microscopy (TEM) images were measured on a JEM-2100F instrument. Elemental analysis was performed in elemental mappings attached to transmission electron microscopy (TEM). X-ray photoelectron spectroscopy (XPS) was recorded on a PerkinElmer PHI 1600 ESCA spectroscope with monochromatic Mg K*α* radiation. UV-vis absorption spectra were conducted by a Hitachi U-3010 spectrometer. Photoluminescence (PL) spectra were recorded on a Jobin Yvon Fluorolog-3 fluorescence spectrometer with excitation at 350 nm.

## Figures and Tables

**Table 1:** Kinetic constants calculated by Equations (4) and (5) with the experimental data of PCN and PCN@TA/PEI-Rh.

Kinetic constants	PCN+free Rh	PCN@TA/PEI-Rh
*k*_1_ (M^−1^ min^−1^)	3.68 × 10^−5^	3.32 × 10^−4^
*k*_2_ (M^−1^ min^−1^)	0.57	12.45
*k*_3_ (M^−1^ min^−1^)	750.00	213.08

## Data Availability

All data used to support the findings of this study are available from the corresponding author upon reasonable request.

## Supplementary Materials

Supplementary Materials Figure S1: schematic reaction mechanism during the preparation of PCN@TA/PEI-Rh core@shell photocatalyst. Figure S2: TEM images of PCN@TA/PEI-Rh (a) and corresponding element mapping of (b) C, (c) N, (d) O, and (e) Rh of PCN@TA/PEI-Rh. Figure S3: XRD patterns of PCN, PCN@TA/PEI, and PCN@TA/PEI-Rh. Figure S4: FT-IR spectra of PCN, PCN@TA/PEI, and PCN@TA/PEI-Rh. Figure S5: high-resolution XPS C 1s spectra of PCN, PCN@TA/PEI, and PCN@TA/PEI-Rh. Figure S6: high-resolution XPS O 1s spectra of PCN, PCN@TA/PEI, and PCN@TA/PEI-Rh. Figure S7: UV-vis diffuse reflectance spectra of PCN, PCN@TA/PEI, and PCN@TA/PEI-Rh. Figure S8: photocurrent responses of PCN and PCN@TA/PEI-Rh at -0.1 V vs. Ag/AgCl. Figure S9: PL spectra of PCN and PCN@TA/PEI-Rh. Figure S10: configuration of (a) bpy-H and (b) bpy-Rh-H. Figure S11: the concentrations of immobilized Rh in different TA and PEI concentration ratios ([TA]/[PEI]). Figure S12: the NADH regeneration efficiency in different coupling reaction time of PEI and bpy-COOH (*X*). Figure S13: the reaction setup of photocatalytic NADH regeneration. Figure S14: recycle stabilities of PCN@TA/PEI-Rh in TEOA solution. Table S1: parameters of time-resolved transient PL described by biexponential function. Table S2: the photocatalytic regeneration performance of NADH by different photocatalysts. Table S3: the experimental data and *r*_NADH_ for photocatalytic NADH regeneration with PCN and PCN@TA/PEI-Rh.
