# Supplementary material for: Intensifying Electron Utilization by Surface-Anchored Rh Complex for Enhanced Nicotinamide Cofactor Regeneration and Photoenzymatic CO2 Reduction
Source: Research (Wash D C). 2021 Feb 18;2021:8175709. doi: 10.34133/2021/8175709 (PMC7910525; doi:10.34133/2021/8175709)
Supplement: Supplementary Materials — Figure S1: schematic reaction mechanism during the preparation of PCN@TA/PEI-Rh core@shell photocatalyst. Figure S2: TEM images of PCN@TA/PEI-Rh (a) and corresponding element mapping of (b) C, (c) N, (d) O, and (e) Rh of PCN@TA/PEI-Rh. Figure S3: XRD patterns of PCN, PCN@TA/PEI, and PCN@TA/PEI-Rh. Figure S4: FT-IR spectra of PCN, PCN@TA/PEI, and PCN@TA/PEI-Rh. Figure S5: high-resolution XPS C 1s spectra of PCN, PCN@TA/PEI, and PCN@TA/PEI-Rh. Figure S6: high-resolution XPS O 1s spectra of PCN, PCN@TA/PEI, and PCN@TA/PEI-Rh. Figure S7: UV-vis diffuse reflectance spectra of PCN, PCN@TA/PEI, and PCN@TA/PEI-Rh. Figure S8: photocurrent responses of PCN and PCN@TA/PEI-Rh at -0.1 V vs. Ag/AgCl. Figure S9: PL spectra of PCN and PCN@TA/PEI-Rh. Figure S10: configuration of (a) bpy-H and (b) bpy-Rh-H. Figure S11: the concentrations of immobilized Rh in different TA and PEI concentration ratios ([TA]/[PEI]). Figure S12: the NADH regeneration efficiency in different coupling reaction time of PEI and bpy-COOH (X). Figure S13: the reaction setup of photocatalytic NADH regeneration. Figure S14: recycle stabilities of PCN@TA/PEI-Rh in TEOA solution. Table S1: parameters of time-resolved transient PL described by biexponential function. Table S2: the photocatalytic regeneration performance of NADH by different photocatalysts. Table S3: the experimental data and rNADH for photocatalytic NADH regeneration with PCN and PCN@TA/PEI-Rh. [file 8175709.f1.doc]

**Supplementary Materials**

**Title**

**Intensifying Electron Utilization by Surface-Anchored Rh-Complex for Enhanced Nicotinamide Cofactor Regeneration and Photoenzymatic CO_2_ Reduction**

**Authors**

Yuqing Cheng^1,3^, Jiafu Shi*^,2,3,4^, Yizhou Wu^1,3^, Xueying Wang^2^, Yiying Sun^1,3^, Ziyi Cai^1,3^, Yu Chen^2^, Zhongyi Jiang*^,1,3^

**Affiliations**

*^1^* Key Laboratory for Green Chemical Technology of Ministry of Education, School of Chemical Engineering and Technology, Tianjin University, 92 Weijin Road, Nankai District, Tianjin 300072, China.

*^2^* School of Environmental Science & Engineering, Tianjin University, 92 Weijin Road, Nankai District, Tianjin 300072, China.

*^3^* Collaborative Innovation Center of Chemical Science and Engineering (Tianjin), 92 Weijin Road, Nankai District, Tianjin 300072, China.

*^4^* State Key Laboratory of Biochemical Engineering, Institute of Process Engineering, Chinese Academy of Sciences, Beijing, 10090, China.

Correspondence should be addressed to Jiafu Shi; [shijiafu@tju.edu.cn](mailto:shijiafu@tju.edu.cn) and Zhongyi Jiang; zhyjiang@tju.edu.cn

**Figure S1.** Schematic reaction mechanism during the preparation of PCN@TA/PEI-Rh core@shell photocatalyst.

**Figure S2.** (a) TEM images of PCN@TA/PEI-Rh and corresponding element mapping of (b) C, (c) N, (d) O and (e) Rh of PCN@TA/PEI-Rh.

**Figure S3.** XRD patterns of PCN, PCN@TA/PEI and PCN@TA/PEI-Rh.

**Figure S4.** FT-IR spectra of PCN, PCN@TA/PEI and PCN@TA/PEI-Rh.

**Figure S5.** High resolution XPS C 1s spectra of PCN, PCN@TA/PEI and PCN@TA/PEI-Rh.

**Figure S6.** High resolution XPS O 1s spectra of PCN, PCN@TA/PEI and PCN@TA/PEI-Rh.

**Figure S7.** UV-vis diffuse reflectance spectra of PCN, PCN@TA/PEI and PCN@TA/PEI-Rh.

**Figure S8.** Photocurrent responses of PCN and PCN@TA/PEI-Rh at -0.1 V vs Ag/AgCl.

**Figure S9.** PL apectra of PCN and PCN@TA/PEI-Rh.


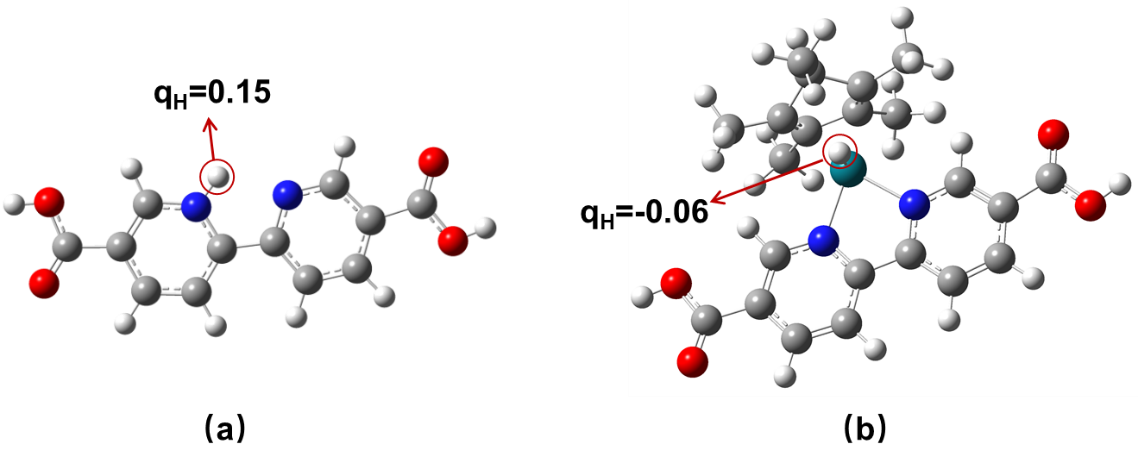


**Figure S10.** Configuration of (a) Bpy-H and (b) Bpy-Rh-H.

**Figure S11.** The concentrations of immobilized Rh in different TA and PEI concentration ratios ([TA]/[PEI]). Reaction conditions: [photocatalyst] = 0.5 mg mL^-1^, volume = 2 mL.

**Figure S12.** The NADH regeneration efficiency in different coupling reaction time of PEI and bpy-COOH (X).


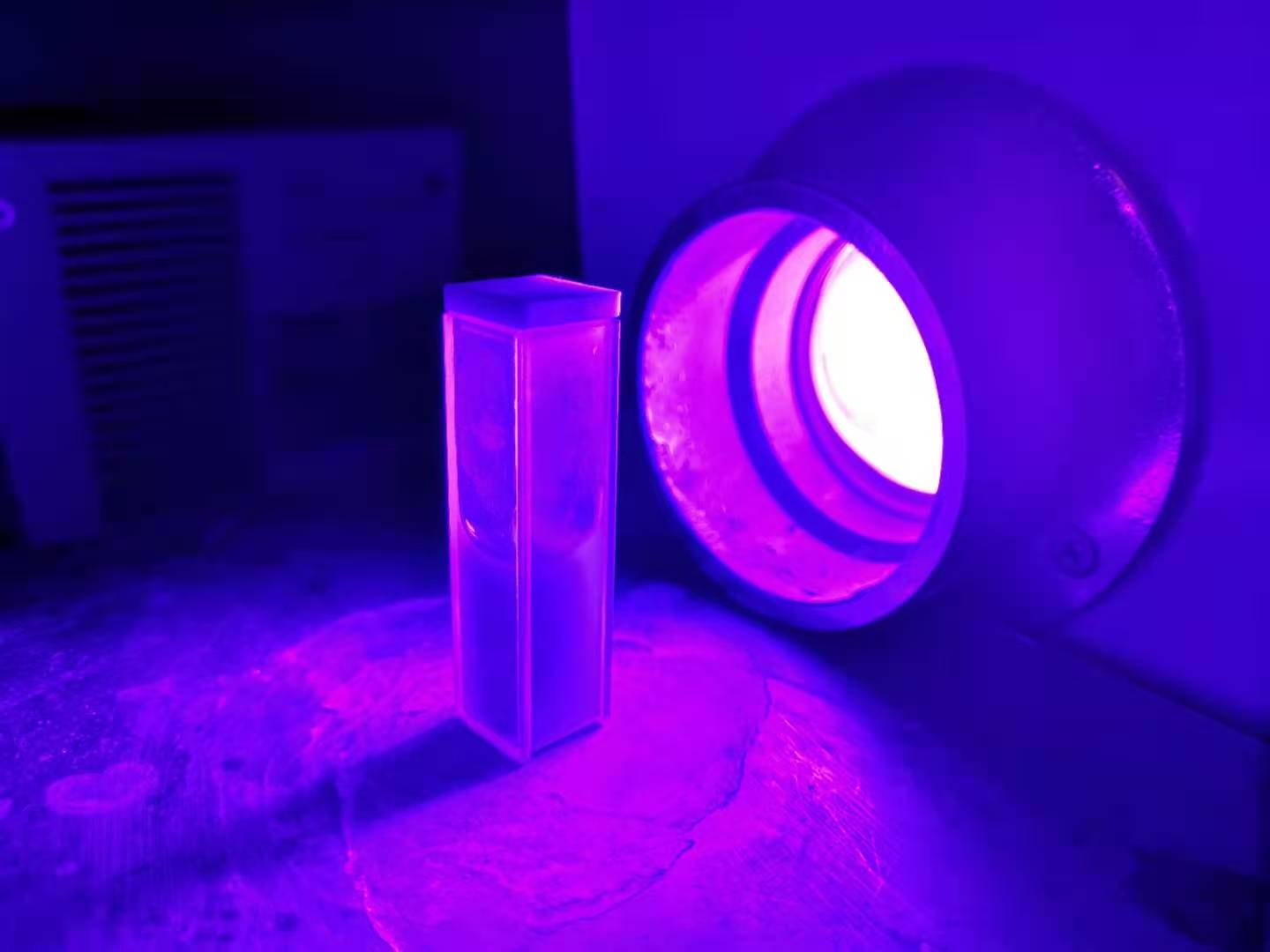


**Figure S13.** The reaction setup of photocatalytic NADH regeneration.


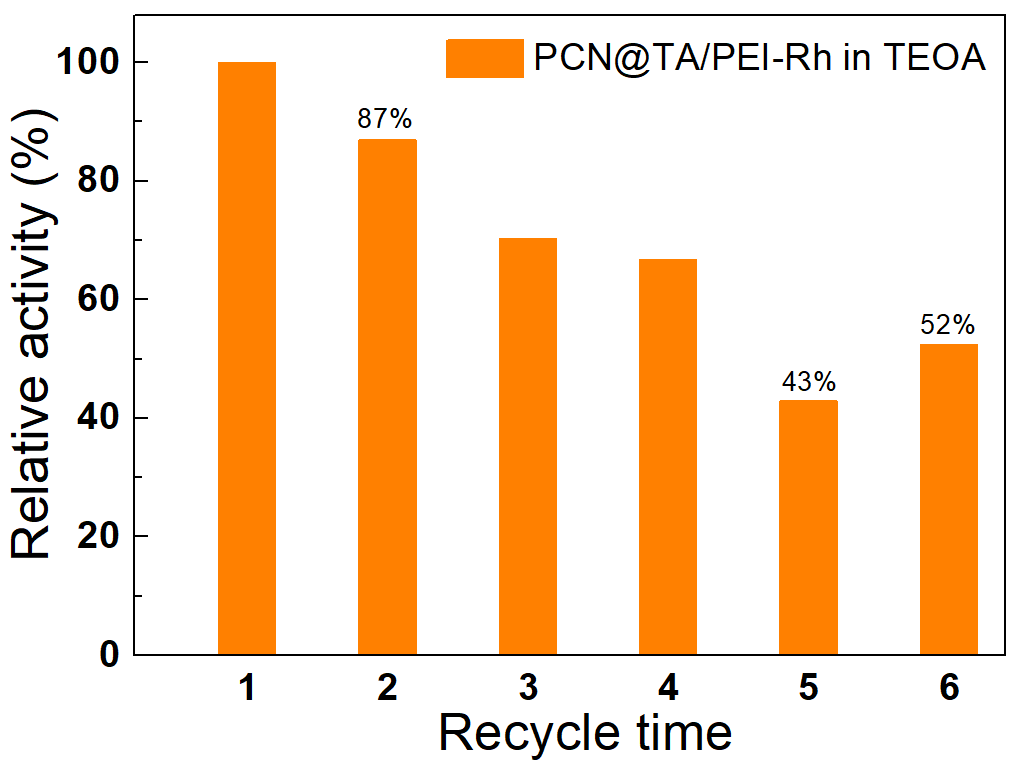


**Figure S14.** Recycle stabilities of PCN@TA/PEI-Rh in TEOA solution.

**Table S1.** Parameters of time-resolved transient PL described by bi-exponential function.

| Photocatalyst | $\tau_{1}$(10^-10^) | $\tau_{2}$(10^-10^) | B_1_(10^-4^) | B_2_(10^-4^) |
| --- | --- | --- | --- | --- |
| PCN | 14.69±0.24 | 102.83±1.13 | 550.33±0.83 | 44.18±0.09 |
| PCN@TA/PEI | 14.76±0.21 | 103.76±1.11 | 559.92±0.83 | 40.85±0.09 |
| PCN@TA/PEI-Rh | 12.81±0.21 | 94.65±1.02 | 600.50±0.92 | 42.55±0.09 |

**Table S2.** The photocatalytic regeneration performance of NADH by different photocatalysts.

| Photocatalyst | Electron donor | Mediator | Balance time (min) | Yield (%) | TOF  (h^-1^) | Refs. |
| --- | --- | --- | --- | --- | --- | --- |
| PCN@TA/PEI-Rh | TEOA | Rh^[a]^ | 20 | 37.8 | 70.82 | Our study |
| g-C_3_N_4_-HTs@rP-QDs | TEOA | Rh | 6 | 80.5 | 46.8 | S1 |
| GCN@a-TiO_2_ | TEOA | Rh | 10 | 82.1 | 19.8 | S2 |
| g-C_3_N_4_@α-Fe_2_O_3_/C | TEOA | Rh | 16 | 76.3 | 22.5 | S3 |
| Rh-NU-1006 | TEOA | Rh | 120 | 28.0 | 20.69 | S4 |
| Co1/C_3_N_4_ | TEOA | Rh | 10 | 98.0 | 33.01 | S5 |
| ATCN-DSCN | TEOA | Rh | 15 | 74.0 | 2.95 | S6 |
| ACN | TEOA | Rh | 60 | 62.3 | 3.36 | S7 |

^[a]^ Rh is [Cp*Rh(bpy)H_2_O]^2+^.

**Table S3.** The experimental data and r_NADH_ for photocatalytic NADH regeneration with PCN and PCN@TA/PEI-Rh.

|  | Catalyst | C_C_  (mM) | TEOA  (mM) | C_Rh_  (mM) | NAD^+^  (mM) | r_NADH_  (mM/min) |
| --- | --- | --- | --- | --- | --- | --- |
| 1 | PCN + free Rh | 5.43 | 400 | 0.028 | 0.1 | 0.00590 |
| 2 | PCN + free Rh | 5.43 | 400 | 0.028 | 0.25 | 0.01045 |
| 3 | PCN + free Rh | 5.43 | 400 | 0.028 | 0.5 | 0.01661 |
| 4 | PCN@TA/PEI-Rh | 5.43 | 400 | 0.028 | 0.1 | 0.00965 |
| 5 | PCN@TA/PEI-Rh | 5.43 | 400 | 0.028 | 0.25 | 0.01391 |
| 6 | PCN@TA/PEI-Rh | 5.43 | 400 | 0.028 | 0.5 | 0.01994 |

**Reaction mechanism and kinetic equations**

The reaction mechanism of photocatalytic NADH regeneration with catalyst was shown below:

where C = Catalyst, C* = Catalyst in its oxidized form, TEOA* = TEOA in its oxidized form, Rh = Rh-complex, Rh-H = Rh-complex in its hydride form.

Therefore, the reaction rate, evaluated in the initial stage, r_NADH_ can be expressed as:

$$r_{NADH}=\frac{d[NADH]}{dt}=k_{3}[Rh-H][NAD^{+}]$$

From the steady-state approximation of Rh and C, the following equations can be obtained:

$$\frac{d[Rh]}{dt}=k_{3}[Rh-H][NAD^{+}]-k_{2}[C][Rh]=0$$

$$\frac{d[C]}{dt}=k_{1}[TEOA][C^{*}]-k_{2}[C][Rh]=0$$

$$k_{3}[Rh-H][NAD^{+}]=k_{1}[TEOA][C^{*}]=0\to[C^{*}]=\frac{k_{3}[Rh-H][NAD^{+}]}{k_{1}[TEOA]}$$

$$[Rh]=\frac{k_{3}[Rh-H][NAD^{+}]}{k_{2}[C]}$$

According to the conservation of mass:

$C_{C}=[C]+[C^{*}]$, $C_{Rh}=[Rh]+[Rh-H]$

$$[Rh]=\frac{k_{3}[Rh-H][NAD^{+}]}{k_{2}\left(C_{C}-[C^{*}]\right)}$$

$$C_{Rh}=\frac{k_{3}[Rh-H][NAD^{+}]}{k_{2}\left(C_{C}-[C^{*}]\right)}+[Rh-H]$$

Finally, the following equation about [Rh-H] can be obtained:

$$k_{1}k_{3}[TEOA][NAD^{+}][Rh-H]=\left(C_{Rh}-[Rh-H]\right)\left(k_{1}k_{2}C_{C}[TEOA]-k_{2}k_{3}[NAD^{+}][Rh-H]\right)$$

**REFERENCES**

1. D. Yang, Y. Zhang, H. Zou et al., “Phosphorus Quantum Dots-Facilitated Enrichment of Electrons on g-C_3_N_4_ Hollow Tubes for Visible-Light-Driven Nicotinamide Adenine Dinucleotide Regeneration,” ACS Sustainable Chemistry & Engneering, vol. 7, no. 1, pp. 285-295, 2019.
2. D. Yang, Y. Zhang, S. Zhang et al., “Coordination between Electron Transfer and Molecule Diffusion through a Bioinspired Amorphous Titania Nanoshell for Photocatalytic Nicotinamide Cofactor Regeneration,” ACS Catalysis, vol. 9, no. 12, pp. 11492-11501, 2019.
3. Y. Wu, J. Ward-Bond, D. Li et al., “g-C_3_N_4_@alpha-Fe_2_O_3_/C Photocatalysts: Synergistically Intensified Charge Generation and Charge Transfer for NADH Regeneration,” ACS Catalysis, vol. 8, no. 7, pp. 5664-5674, 2018.
4. Y. Chen, P. Li, J. Zhou et al., “Integration of Enzymes and Photosensitizers in a Hierarchical Mesoporous Metal-Organic Framework for Light-Driven CO_2_ Reduction,” Journal of the American Chemical Society, vol. 142, no. 4, pp. 1768-1773, 2020.
5. W. Liu, W. Hu, L.Yang et al., “Single Cobalt Atom Anchored on Carbon Nitride with Well-Defined Active Sites for Photo-Enzyme Catalysis,” Nano Energy, vol. 73, pp. 104750, 2020.
6. J. Meng, Y. Tian, C. Li et al., “A Thiophene-Modified Doubleshell Hollow g-C_3_N_4_ Nanosphere Boosts NADH Regeneration via Synergistic Enhancement of Charge Excitation and Separation,” Catalysis Science & Technology, vol. 9, no. 8, pp. 1911-1921, 2019.
7. E. J. Son, Y. W. Lee, J. W. Ko et al., “Amorphous Carbon Nitride as a Robust Photocatalyst for Biocatalytic Solar-to-Chemical Conversion,” ACS Sustainable Chemistry & Engineering, vol. 7, no. 2, pp. 2545-2552, 2019.
